# Causative Genes of Homologous Recombination Deficiency (HRD)-Related Breast Cancer and Specific Strategies at Present

**DOI:** 10.3390/curroncol32020090

**Published:** 2025-02-06

**Authors:** Seigo Nakamura, Yasuyuki Kojima, Sayoko Takeuchi

**Affiliations:** 1 Institute for Clinical Genetics and Genomics, Showa University, Tokyo 142-8555, Japan; yasucozy_8@med.showa-u.ac.jp (Y.K.); sayocgg@med.showa-u.ac.jp (S.T.); 2 Division of Breast Surgical Oncology, Department of Surgery, Showa University, Tokyo 142-8666, Japan

**Keywords:** HRD-related genes, *BRCA*, *PARP* inhibitor

## Abstract

Recently, homologous recombination deficiency (HRD) has become a new target for hereditary cancers. Molecular-based approaches for hereditary cancers in the clinical setting have been reviewed. In particular, the efficacy of the *PARP* inhibitor has been considered by several clinical trials for various kinds of hereditary cancers. This indicates that the *PARP* inhibitor can be effective for any kind of *BRCA* mutated cancers, regardless of the organ-specific cancer. Homologous recombination deficiency (HRD) has become a new target for hereditary cancers, indicating the necessity to confirm the status of HRD-related genes. *ARID1A*, *ATM*, *ATRX*, *PALB2*, *BARD1*, *RAD51C* and *CHEK2* are known as HRD-related genes for which simultaneous examination as part of panel testing is more suitable. Both surgical and medical oncologists should learn the basis of genetics including HRD. An understanding of the basic mechanism of homologous repair recombination (HRR) in *BRCA*-related breast cancer is mandatory for all surgical or medical oncologists because *PARP* inhibitors may be effective for these cancers and a specific strategy of screening for non-cancers exists. The clinical behavior of each gene should be clarified based on a large-scale database in the future, or, in other words, on real-world data. Firstly, HRD-related genes should be examined when the hereditary nature of a cancer is placed in doubt after an examination of the relevant family history. Alternatively, HRD score examination is a solution by which to identify HRD-related genes at the first step. If lifetime risk is estimated at over 20%, an annual breast MRI is necessary for high-risk screening. However, there are limited data to show its benefit compared with *BRCA*. Therefore, a large-scale database, including clinical information and a long-term follow-up should be established, after which a periodical assessment is mandatory. The clinical behavior of each gene should be clarified based on a large-scale database, or, in other words, real-world data.

## 1. Introduction

For the past three decades, hereditary breast and ovarian cancer (HBOC) has been a major topic for breast surgeons [1,2].

*BRCA* genes are a subset of homologous recombination deficiency (HRD)-related genes, which are associated with an increased risk of developing breast, ovarian, and other cancers. The *BRCA1* and *BRCA2* genes code for proteins that play a critical role in DNA repair, specifically in the process of homologous recombination. When these genes are mutated or altered, cells become more susceptible to DNA damage and can acquire additional mutations that may lead to the development of cancer. 

Individuals with inherited mutations in *BRCA1* or *BRCA2* have a significantly higher risk of developing breast and ovarian cancers compared with the general population. The estimated cumulative risks of breast cancer by age 70 in two meta-analyses were 55% to 65% for carriers of *BRCA1* pathogenic variants and 45% to 47% for carriers of *BRCA2* pathogenic variants [3]. In addition, these mutations are associated with an increased risk of developing other types of cancers, such as pancreatic and prostate cancers. The cumulative risks to those at age 80 are reported as being approximately 2.5% for pancreatic cancer for both *BRCA1* and *BRCA2* carriers, and 27% for prostate cancer for *BRCA2* carriers [4].

Surveillance procedures and intervals for *BRCA* mutation carriers is well summarized in the NCCN guideline. At age 18, it is recommended to develop breast awareness, and a clinical breast exam is recommended every 6 to 12 months for those at the age of 25 years. Above the age of 25, annual breast MRI surveillance is recommended [5,6,7,8,9].

Furthermore, risk reduction mastectomy (RRM) or risk reduction salpingo-oophorectomy (RRSO) should be considered for pathogenic *BRCA* mutation careers [10,11].

Overall survival was longer with RRSO compared with no RRSO (HR 0.32, 95% CI 0.19 to 0.54; *p* < 0.001 [11]). The efficacy of those prophylactic surgeries has been proven through several case-control studies [12] and are recommended in clinical practice guidelines worldwide [13,14]. 

The pathogenic alteration of *BRCA1/2* has several prophylactic procedures by which to develop a feeling of benefit to each client.

*BRCA* testing was almost never undertaken in Asian countries in the past 20 years; however, it has become very popular at present and the ratio of hereditary breast cancers has been found to be almost the same [15,16]. 

These days, the value of genetic testing without *BRCA1/2* has gradually become clarified in Asia [17,18,19,20,21,22,23,24,25]. 

The homologous recombination repair system is a key pathway for repairing double-stranded DNA breaks and is essential for maintaining genomic stability. HRD-related genes play a crucial role in this process.

Mutations in several HRR systems can lead to HRD and impaired DNA repair, which can increase the risk of cancer and affect the response to certain cancer therapies. Some of the most well-known HRD-related genes include *BRCA1*, *BRCA2*, *RAD51* (*Radiation Sensitive 51*), *ATM*, and *PALB2*. These genes are often associated with hereditary breast and ovarian cancer syndromes, as well as other cancer types. *TP53* and *PTEN* can also cause hereditary breast cancer, but they are less common than *BRCA1* and *BRCA2* mutations. Moreover, the presence of variants of unknown significance (VUS) is still higher than in *BRCA1/2.* Long-term follow-up of these cancers and the maintenance of their databases are very important to the understanding of their pathogenicity.

HRD testing is becoming an increasingly important tool in cancer treatment decision-making, particularly for patients with breast, ovarian, and prostate cancers. Testing for HRD status can help identify patients who may benefit from certain targeted therapies, such as those associated with *PARP* inhibitors. The mechanism of resistance to *PARP* inhibitors has been considered and a newer type of *PARP* inhibitor has been developed. Combination therapy with other agents has been verified under clinical trials. The scope of *PARP* inhibitors in the near future will be mentioned in this review.

## 2. Multi-Gene Panel Testing for Hereditary Breast Cancer

Nowadays, there are several other genes which cause hereditary breast cancer that have been found by multi-gene panel testing [11,26,27]. The genes related to the penetrance of breast cancer are divided into three groups (high, moderate and low). The high-risk genes are *BRCA1/2, PALB2, P53, PTEN, CDH1* (*Cadherin 1*) and *STK11*. They have an above 50% lifetime probability of breast cancer occurrence. 

The moderate risk genes are *ATM, CHEK2, BRIP1* and *RAD51*. They have an above 20% lifetime risk of breast cancer occurrence and surveillance using breast MRI is recommended; however, RRM is thought to be an overtreatment for this in the new guideline.

*P53* is a tumor suppressor protein that plays a critical role in preventing the development and progression of cancer. It acts as a checkpoint, detecting damaged DNA and either triggering DNA repair or inducing cell death to prevent the propagation of damaged DNA. Mutations in the *P53* gene that encodes for the *P53* protein are associated with an increased risk of cancer development and progression, as well as resistance to chemotherapy and radiation therapy. Strategies to restore or enhance *P53* activity are currently being explored as potential cancer therapies [28].

The phosphatase and Tensin homolog (*PTEN*) is a gene that produces a protein that helps regulate the growth and division of cells in the body. The *PTEN* gene is found on chromosome 10 and is a tumor suppressor gene. Mutations in the *PTEN* gene have been linked to the development of a variety of cancers, including breast, prostate, and thyroid cancer, as well as Cowden syndrome, a genetic disorder characterized by multiple tumor growths in various parts of the body. The *PTEN* gene is also involved in many biological processes beyond cancer, such as cell migration, cell survival, and response to stress. Therefore, scientists continue to study the role of the *PTEN* gene in order to gain a greater understanding of its impact on human health [29]. *PTEN* mutations may overlap with other mutations, including human epidermal growth factor receptor 2 (HER2) and loss of a single *PTEN* allele has been shown to accelerate tumorigenesis in HER2-overexpressing breast tumors [30]. *PTEN* loss and *PTEN*-independent activation of the *PI3K* pathway have been identified as major determinants of trastuzumab resistance in preclinical models and also in clinical samples [31].

The *CDH1* gene codes for a protein called E-cadherin, which is involved in cell adhesion in tissues. E-cadherin is a protein that helps cells stick together to form tissues and organs. It is particularly important in epithelial cells, which line the surfaces of organs and structures within the body, such as the skin and the linings of the lungs, liver, and stomach. Mutations in the *CDH1* gene have been associated with a higher risk of certain types of cancer, particularly stomach (gastric) cancer and lobular breast cancer, which is known as hereditary diffuse gastric cancer syndrome. Histologically, this syndrome is characterized by the diffuse presence of signet ring cells and poorly connective carcinoma cells with in situ or pagetoid features in the gastric mucosa. Testing for mutations in the *CDH1* gene may be recommended for individuals with a personal or family history of these types of cancer [32]. In a recent cohort study of 394 women with lobular breast carcinoma (LBC), 15 germline *CDH1* variants were identified in 15 families with hereditary lobular breast carcinoma (HLBC); 40.0% were pathogenic or likely pathogenic (P/LP). The overall frequency of P/LP *CDH1* variants was 1.5% and was associated with age of 45 years or younger at LBC diagnosis and positive family history of BC. Therefore, the identification of P/LP germline *CDH1* variants in young women with LBC with (or without) family history of BC, not fulfilling the classic *CDH1* genetic screening criteria, may provide an indication to test for *CDH1* gene [33].

*STK11* is a serine/threonine kinase that regulates cell polarity and energy metabolism and functions as a tumor suppressor. Mutation in this gene is well known as a cause of *PTEN* mutations that may overlap with other mutations, including human epidermal growth factor receptor 2 (HER2) and loss of a single PTEN allele has been shown to accelerate tumorigenesis in HER2-overexpressing breast tumors, Peutz–Jeghers syndrome, as well as with skin, pancreatic, and testicular cancers.

**Figure 1:**
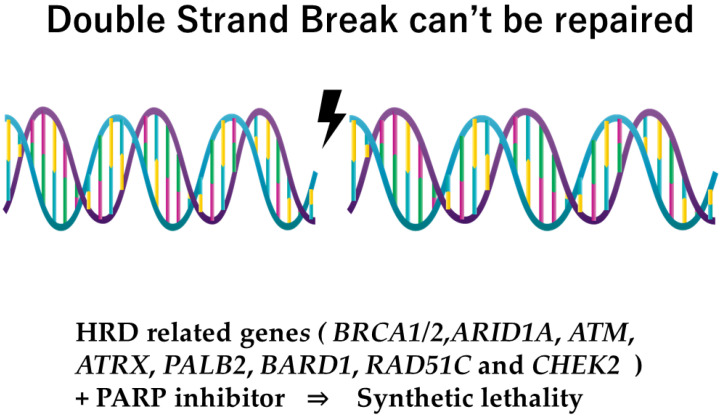
HRD-related genes and *PARP* inhibitor.

Homologous recombination deficiency (HRD) has become a new target for hereditary cancers, which indicates the necessity of confirming the status of HRD from the aspect of the efficacy of the *PARP* inhibitor. *ARID1A*, *ATM*, *ATRX*, *PALB2*, *BARD1*, *RAD51C* and *CHEK2* are known as HRD-related genes. Some of these have already shown efficacy with regard to the PARP inhibitor (Figure 1).

*PALB2* is a gene that stands for Partner and Localizer of BRCA2. It is associated with an increased risk of breast and ovarian cancer. Mutations in the *PALB2* gene can disrupt the normal functioning of proteins involved in DNA repair, which can lead to a higher risk of developing cancerous cells.

The *PALB2* gene is often tested in individuals with a family history of breast or ovarian cancer, especially those with a known *BRCA1* or *BRCA2* mutation. It is important to consult with a medical professional or genetic counselor for more detailed information and guidance regarding genetic testing and screening [34].

The Ataxia Telangiectasia Mutated (*ATM*) gene is a gene that codes for a protein involved in cell cycle regulation and DNA repair. Mutations in this gene can cause a rare genetic disorder known as Ataxia telangiectasia (A-T), which is characterized by neurological symptoms, a weakened immune system, and an increased risk of developing certain types of cancer. The protein produced by the *ATM* gene helps to activate cell cycle checkpoints in response to DNA damage, allowing time for DNA repair to occur before the cell continues to divide. In individuals with A-T, the *ATM* protein is either absent or not functional, which can lead to an accumulation of DNA damage and an increased risk of developing cancer. People with mutations in the *ATM* gene may also have an increased sensitivity to radiotherapy and certain chemotherapeutic agents used in cancer treatment [35,36,37].

Checkpoint kinase 2 (*CHEK2*) is a gene that codes for a protein involved in cell cycle regulation and DNA repair. It is located on chromosome 22q12.1 and is also known as *CHEK2*. Mutations in the *CHEK2* gene have been linked to an increased risk of developing certain types of cancer, including breast, prostate, and colorectal cancer. Individuals with mutated *CHEK2* have an increased risk of developing cancer at a young age and may benefit from increased cancer surveillance and preventative measures. Testing for *CHEK2* mutations may be recommended for individuals with a family history of cancer or other risk factors [38,39]. 

The *RAD51* gene codes for a protein called *RAD51*, which plays a crucial role in DNA repair. *RAD51* is involved in homologous recombination, which is a process by which damaged DNA strands are repaired using the information from an undamaged duplicate copy of the DNA. Homologous recombination is an essential DNA repair mechanism that helps prevent the accumulation of mutations and the development of cancer. Mutations in the *RAD51* gene have been linked to an increased risk of breast, ovarian, and other cancers [39]. 

The *BRIP1* gene, also known as *BRCA1*-Interacting Protein C-terminal Helicase 1, is involved in DNA repair and maintenance of genomic stability. It is important for individuals with a family history of breast or ovarian cancer to consider genetic testing for *BRIP1* mutations in order to assess their risk and make informed medical decisions [40]. Long-term follow-up is warranted to judge what kind of prophylactic strategy is appropriate for *BRIP1*.

The cost of genetic testing has become less expensive and the results are more rapidly obtained. Therefore, long-term follow-up data are warranted in order to judge what kind of prophylactic strategy is appropriate for reasonable multi-gene panel testing [41,42,43,44,45]. The lifetime risk of breast cancer caused by each gene has gradually been clarified.

However, the other genes, beyond BRCA1/2, which are supposed to be the cause of hereditary breast cancer have a much higher frequency of variants of uncertain significance (VUS) [46].

Long-term follow up and the development of an associated database are warranted to clarify the necessity of RRM or breast MRI [47,48]. 

The new guideline for ASCO, stated in January 2024, is as follows:

- Offer *BRCA1/2* testing to all patients diagnosed with breast cancer at or below age 65.
- Testing for other high penetrance genes, such as *PALB2*, *TP53*, *PTEN*, *STK11*, and *CDH1,* should be offered to appropriate patients, as mutations in these genes could inform medical treatment, influence surgical decision making, refine estimates of second primary cancer risks, and inform family risk assessment.
- Testing for moderate penetrance genes, such as *ATM, CHEK2, RAD51C, RAD51D* and *BARD1*, may be offered to appropriate patients who are undergoing BRCA1/2 testing.
- While mutations in these genes may inform the risks of second primary cancer or family risk assessment, they currently offer no treatment benefits for breast cancer patients.
- Nowadays, the genes relating to homologous repair recombination (HRR) divide into one group, known as homologous repair deficiency (HRD), that refers to a dysfunction of HRR, or, in other words, to a group for which the PARP inhibitor will have the possibility of efficacy.

Recently, multi-gene panel testing has become more common and the etiological data associated with them has gradually been collected, one of the solutions to collect the worldwide data is the I-CARE project conducted by a group at Vanderbilt university. I-CARE is a registry of individuals interested in participating in inherited cancer research, through which data and samples are collected to contribute to research. Participants are also provided with ongoing research and clinical updates and informed about other research opportunities for which they might be eligible. Participants are recruited from all over the world. There is no cost to participate, and all materials can be completed online. 

One of the studies this group has conducted was focused on *PALB2, ATM,* and *CHEK2* mutations in order to evaluate breast cancer treatment and characteristics by conducting additional tumor genomic studies [49].

## 3. Poly (Adenosine Diphosphate-Ribose) Polymerase Inhibitors (PARP Inhibitor) for Hereditary Breast Cancer

Synthetic lethality is a genetic concept that describes a situation in which two mutations, each of which is not lethal by itself, cause cell death when combined. *PARP* inhibitors (*PARP*is) are drugs that exploit synthetic lethality to target cancer cells that have defects in DNA repair pathways, such as those caused by mutations in BRCA1 or BRCA2 genes. *PARP*is block the activity of *PARP* enzymes, which are involved in repairing single-strand DNA breaks (SSBs). When *PARP*is are used in cells that have normal homologous recombination (HR) repair, the SSBs can be fixed by HR. However, when *PARP* inhibitors are used in cells that have impaired HR repair, the SSBs accumulate and become double-strand breaks (DSBs), which are more lethal and difficult to repair. This leads to genomic instability and cell death in the cancer cells, while sparing the normal cells [50].

*PARP* inhibitors have been used for metastatic breast cancers for several years [51,52]. 

*PARP* inhibitors are a class of drugs that target the PARP enzymes in cells, which play a critical role in DNA repair. These inhibitors work by blocking the activity of *PARP*, which leads to the accumulation of DNA damage and ultimately triggers cell death in cancer cells.

*PARP* inhibitors have become a game-changer in cancer treatment, particularly in cancers with *BRCA* mutations, which are more susceptible to DNA damage. Clinical trials have shown impressive results in ovarian, breast, prostate, pancreatic, and other cancers, where *PARP* inhibitors have led to prolonged survival rates [53,54,55,56,57].

The use of *PARP* inhibitors in combination with other treatments, such as chemotherapy and immunotherapy, is also being explored as a way by which to increase treatment effectiveness and overcome drug resistance.

However, like all treatments, *PARP* inhibitors can have side effects, including nausea, fatigue, and anemia. Patients should discuss the risks and benefits of *PARP* inhibitors with their healthcare providers.

Overall, *PARP* inhibitors are a promising new class of cancer treatments that provide hope and new options for patients with difficult-to-treat cancers. 

Recently, Olaparib, has become a *PARP* inhibitor added as part of a standard regimen for *BRCA1* or *BRCA2* germline pathogenic, or likely pathogenic, variants and high-risk clinicopathological factors which had received local treatment and neoadjuvant or adjuvant chemotherapy [58].

The long-term effect of adjuvant Olaparib is interesting in terms of the necessity of RRM or RRSO. The scope of Olaparib has become wide enough to include metastatic pancreatic cancer and metastatic castration-resistant prostate cancer, although its approval status varies by country; for example, in Italy, Olaparib is no longer subsidized for patients with metastatic pancreatic cancer. This means that *PARP* inhibitors can be effective for *BRCA* mutated cancers regardless of the organ-specific cancer.

HRD has become a new target for metastatic cancers, which indicates the necessity of confirming the status of HRD-related genes [59,60,61,62,63], with *ARID1A, ATM, ATRX, PALB2, BARD1, RAD51C* and *CHEK2* being the HRD-related genes for which simultaneous examination as part of panel testing has been shown to be more suitable [64,65].

Confirming the status of HRD using cancerous tissue is becoming the standard when determining the indication of a PARP inhibitor in ovarian cancer [66,67,68].

In recent years, several mechanisms of resistance have been reported. One of the mechanisms of resistance of PARP inhibitors, the restoration of function by *BRCA1/2* secondary mutation (reversion mutation), has attracted attention [69]. Reversion mutation has been reported to occur in various kinds of cancers and its solution is an urgent matter at present [70]. Secondly, replication fork stabilization is another cause of resistance, one which can be caused by proteins like the Fanconi anemia group D2 (FANCD2) protein, SNF2-Related Chromatin Remodeling Annealing Helicase (*SMARCAL1*), Helper T-Lymphocyte Frequency (HTLF), and Zinc Finger RANBP2-Type Containing 3 (ZRANB3). Thirdly, loss of the 53BP1–RIF1–REV7–Shieldin axis, which reactivates resection and homologous recombination (HR) in *BRCA1*-deficient cells is thought to be another reason for resistance. Lastly, increased drug efflux, which is caused by overexpression of P-glycoprotein (P-gp) efflux pumps and which is often seen during conventional chemotherapy, is also a cause of resistance.

Research on resistance mechanisms to PARP inhibitors is ongoing. Some ongoing trials, such as the NEO study and ARIEL 2, are seeking to identify predictors of response and overcome resistance to PARP inhibitors. These trials have shown promising results at present. On the other hand, a partner trial addition of Olaparib to neoadjuvant chemotherapy to TNBC with BRCA wild type has shown no benefit.

## 4. Surveillance for Other Cancers

Unaffected male cancers can be found by triggering BRCA-mutated breast cancer in patients [71].

Because there are high-risk forms of prostate cancer, annual PSA monitoring should be used for its early detection [72], as should magnetic resonance cholangio-pancreatography (MRCP). There is a research program which can adapt an MRI screening protocol for pancreatic cancer, which is to be performed in conjunction with breast MRI screening in BRCA positive individuals [73,74]. Therefore, the role of genetic counsellors has become more important than ever when seeking to refer each individual to a proper department at the right time. 

In one case-control study of 63,828 patients with 14 common cancer types and 37,086 controls in Japan, pathogenic variants in *BRCA1* were associated with biliary tract cancer, in *BRCA2* with esophageal cancer, and in *BRCA1/2* with gastric cancer [75]. This means that annual surveillance for the gastrointestinal tract might be considered for *BRCA1* or *2* mutated cancers [76].

## 5. Risk -Stratified Screening for Breast Cancer in the Future

As previously stated, multi-gene panel testing will soon become the standard. Additionally, the testing criteria will become wider and cover the other hereditary cancers [70,72,73,75,77,78].

Recently, polygenic risk scores (PRSs) have been used to stratify women according to their risk of developing primary invasive breast cancer [79,80,81]. Several studies are ongoing at present. Risk-stratified screening for breast cancer is anticipated in the future [82,83,84].

## 6. Significance of Multi-Disciplinary Approach

*BRCA1/2* pathogenic mutation causes not only breast or ovarian cancer but also prostate or pancreatic cancer. Therefore, periodic communication between multiple fields is necessary and the key person for multi-disciplinary communication is the genetic counselor or advanced nurse practitioner [85,86,87,88,89,90,91].

## 7. Conclusions

An understanding of the basic mechanism of HRR in *BRCA*-related breast cancer is mandatory for all surgical or medical oncologists because *PARP* inhibitors may be effective for cancers and for specific strategies of screening for non-cancers. In the future, the clinical behavior of each gene should be clarified based on large-scale databases, or, in other words, real-world data.

**Figure 2:**
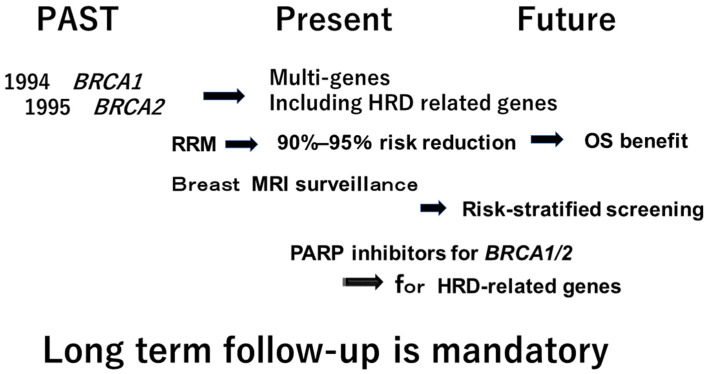
Past, Present and Future of hereditary breast cancer.

Firstly, HRD-related genes should be examined in cases when the hereditary nature of a cancer is cast in doubt through the examination of the relevant family history. Alternatively, HRD score examination can be used to identify HRD-related genes at the first step. If lifetime risk is estimated to be over 20%, annual breast MRI is necessary as a form of high-risk screening. However, there are limited data to show its benefits when compared with *BRCA*. Therefore, a large-scale database, including clinical information, is mandatory (Figure 2).
